# From Local Checklists to Online Identification Portals: A Case Study on Vascular Plants

**DOI:** 10.1371/journal.pone.0120970

**Published:** 2015-03-19

**Authors:** Stefano Martellos, Pier Luigi Nimis

**Affiliations:** Department of Life Sciences, University of Trieste, via L. Giorgieri 10, Trieste, Italy; Consiglio Nazionale delle Ricerche (CNR), ITALY

## Abstract

Checklists, the result of time-consuming exploration and painstaking bibliographic research, can be easily converted into online databases, which have the advantage of being updatable online in real time, and of reaching a much wider audience. However, thousands of local checklists (Natural Parks, protected areas, etc.) are still available on paper only, and most of those published online appear as dry lists of latin names, which strongly reduces their outreach for a wider audience. The University of Trieste has recently started the publication of several local checklists in a way that may be more appealing for the general public, by linking species' names to archives of digital resources, and especially to digital identification tools produced by software FRIDA (FRiendly IDentificAtion). The query interfaces were developed on the basis of feedback from a wide range of users. The result is no longer a simple list of names accessible on the Web, but a veritable multimedial, interactive portal to the biodiversity of a given area. This paper provides an example of how relevant added value can be given to local lists of taxa by embedding them in a complex system of biodiversity-related resources, making them usable for a much wider audience than a restricted circle of specialists, as testified by the almost 1.000.000 unique visitors reached in 2014. A critical mass of digital resources is also put at disposal of the scientific community by releasing them under a Creative Commons license.

## Introduction

A checklist, which summarises the hitherto known biological diversity of a territory for a given group of organisms, is the result of careful and thorough field work, as well as of time-consuming bibliographic research. The compilation of a checklist is a kind of “never-ending story”, because further exploration may lead to the discovery of new taxa, often rendering a checklist outdated in a few years. In the literature there are thousands of local checklists referring to relatively small areas (e.g. a Natural Park, a protected area, a mountain, etc.) which are available in paper-form only, either as a book [1, 2], or as an article in the bulletins of Scientific Societies, Museums, and Universities [3, 4]. Although some open-access journals, thanks to their online platforms, have recently demonstrated some interest in publishing checklists [5], these rarely find a space in indexed scientific journals, mainly because of their bulky size. Rendering these data available online could give them a consistent added value.

Checklists fit well a conversion into online databases [6], because they are simple lists of names, to which other information is associated (e.g. synonyms, critical annotations, occurrence records). While updating a book or an article does require a new edition—see as an example the updates of the Checklist of the Italian Vascular Flora [7], published by the Museum of Vicenza [3]—an online database can be updated in real time, or at more or less regular intervals. An example is the Plants of Southern Africa Database (http://posa.sanbi.org) [8], which is updated every two months. In the last decades, several checklists of large areas have been published online, with different formats and degrees of interactivity, and of complexity. Some examples are: the annotated checklist of the flowering plants of Nepal (http://padme.rbge.org.uk/floraofnepal/); the Euro+Med PlantBase [9], or VICTORIA, an on-line information system on the lichens of Victoria Land [10]. However, most of the checklists published online appear as dry lists of latin names, which strongly reduces their outreach for a wider audience

The Department of Life Sciences of the University of Trieste is working since 2002 in the publication of biodiversity data on the Web. The first products published online were national checklists, e.g. of the lichens [1, 11, 12], mosses [13] and macrobasidiomycetes of Italy [2]. All of them, because of their importance in research and education, are now integrated into the Italian National Biodiversity Network [14, 15].

A second step was that of creating a series of interactive keys for local floras, or for certain groups of organisms (mosses, algae, lichens, microfungi, butterflies, fishes, marine organisms etc.), produced by software FRIDA (FRiendly IDentificAtion) [16, 17]. The more than 600 dichotomous keys generated by FRIDA differ from most 'classical' keys in being completely independent from biological systematics. They do not start with a key to supra-specific taxa, which are usually distinguished by 'difficult' characters [16, 18], and hence are usable also by a non-specialised audience.

The third step, which is illustrated in this paper, was that of embedding identification tools into local checklists published online, with the aim of opening their content to a wider audience, which can access the information for curiosity, interest in biodiversity, professional needs, pedagogical scenarios, decision-making, etc. Previous experiences, such as those acquired in the European projects KeyToNature [19], VIBRANT [20], Open Discovery Space (http://opendiscoveryspace.eu/) and SiiT (http://www.siit.eu/), convinced us that a much wider audience can be reached only if the needs of potential users are properly analysed and addressed, an approach which is rarely used in the case of biodiversity-related online databases. The possibility of automatically transforming a local checklist into a portal including also an identification tool was a challenging task, involving an understanding of principles, technologies, domain knowledge and terminology, as well as pedagogical skills. We had to address issues related to the usability of online biodiversity resources by analysing feedback from users, and to customise the query systems and interfaces accordingly, to render them more user-friendly and appealing for a wider audience. The result is no longer a simple list of names accessible on the Web, but a veritable multimedia, interactive portal to the biodiversity of a given area. The success of these products among users is testified by the almost 1.000.000 unique visitors reached in 2014.

This paper aims at describing our experience in publishing local checklists online, as an example of how relevant added value can be given to local lists of taxa by embedding them in a complex system of biodiversity-related resources, making them usable for a much wider public than a restricted circle of specialists. A critical mass of digital resources is also put at disposal of the scientific community by releasing them under a Creative Commons license.

## Feedback from Users

The portals described in this paper have been developed on the basis of the expertise acquired during years of research on biodiversity-related digital systems. Feedback was collected from a wide range of users, differing in age, skills, background and interests, on the basis of interviews, questionnaires and Focus Groups in the framework of the European Projects KeyToNature (2007–2010) and SiiT (2011–2014).

The first test was dedicated to the performance of the dichotomous keys produced by FRIDA as compared with 'classical', paper-printed keys. Sixty university students (first year of Biology), divided into 2 groups of 30, were asked to identify 15 plant species: the first group could use any among three 'classical' keys [21, 22, 23], the second group used the interactive dichotomous key to the Flora of the Rosandra Valley near Trieste (ca. 1000 species), produced by software FRIDA. Average time for the identification and the percents of misidentifications were recorded.

The second test concerned the usability and the psychological impact of different query interfaces: dichotomous, multi-entry (several characters among a limited list can be combined in the first step of the identification process), and free access (users can choose from a comprehensive list which character to use in each step of the identification process) [24]. This test was carried out with several Focus Groups comprising altogether 676 persons, subdivided by level of expertise: 1) elementary school childrens, 2) High school students, 3) University students of Biology (master), 4) University students of Biology (PhD), 4) University Professors of Botany, 5) General Public (volunteers, excluding students and teachers). Every group was asked to identify 5 plants using 3 different query interfaces produced by FRIDA for the Val Rosandra Flora: (dichotomous, free access, multi-entry), to vote for the preferred interface, and to justify their choice in the form of a note.

The results of these tests, and of more than 25.000 questionnaires received from school teachers across Europe, brought to a radically new design of the query interfaces for the portals created around a local checklist. The results can be summarised as follows:

1) The use of the dichotomous keys produced by FRIDA results in a much shorter identification time, and in a much lower number of misidentifications (Table 1).

**Table 1:** Performance of a digital identification key produced by software FRIDA (group A) as compared with 'classical' paper-printed keys (group B).

	Group A	Group B
	Digital identification keys	‘Classical keys’
Total time (minutes)	52	194
Avg. time per species (minutes)	3.5	12.9
Percentage of misidentification	12	46

Each group consisted of 30 university students; both groups had to identify 15 species. Total time, average time per identification, and percentage of misidentification are reported.

2) The dichotomous interface is preferred by all users' groups, although interest in the multi-entry interface and the free-access interface [24] increases with the level of expertise. (Table 2). The dichotomous interface is perceived as the most user-friendly, and the most effective in identifying closely related taxa, especially in critical groups where species are often distinguished by a combination of characters rather than by a single character. Its main drawback is evident when a specimen lacks one or more characters whose observation is required.

**Table 2:** Preference of different categories of users among three different types of identification interfaces (in percentages).

	General public	Primary school	Secondary schools	University (master)	University (PhD)	Specialists
N° of users	122	258	62	137	65	32
Avg. age	23–74	8	15	20	25	52
Dichotomous	86	97	89	72	63	50
Multi-entry	10	3	8	16	19	26
Free access	4	0	3	12	18	24

3) The free-access interface is perceived as the most unfriendly, users being often confused by the high number of available characters to choose from; it was also found rather poor for distinguishing among closely related taxa; however, the opportunity of having more freedom in the choice of characters was seen as an important advantage by several users.

4) The multi-entry interface is appreciated for greatly reducing the number of remaining taxa in a single step, although it does not always permit to achieve an identification at species level, especially in the case of large floras.

While building our portals, we tried to transfer the input from users into a new and effective product, by combining the effectiveness of multi-entry interfaces in reducing the list of taxa with the higher performance of dichotomous keys in dealing with closely related taxa belonging to critical groups. The dichotomous key is invoked only for the species which have been previously selected with the multi-entry interface, which greatly reduces the number of passages required for an identification. The graphic outlines of both the multi-entry and the dichotomous interfaces were also designed and tested on the basis of feedback from users.

## Data and Methods

### Creation of a portal

A pre-requisite for the creation of a portal is the availability of an online reference checklist, usually already published in paper-form, which automatically provides a solid nomenclatural and taxonomical background, also including a thesaurus of synonyms. In Italy, as far as vascular plants are concerned, we use a digital version of the checklist of the flora of Italy [7], updated to 2012 [3]. Another pre-requisite is the availability of a good local checklist of taxa occurring in a given area.

A portal is made of a core, i.e. the software which permits the queries and displays the results, and of several connections to other databases/archives which were developed and published online since 2002. Hence, a portal has a distributed architecture, in which relevant information and media are obtained from online resources when required. The core of a portal is made of a MySQL database storing the morphological, distributional and nomenclatural data which are used in the query system, written in PHP 5.0. The interfaces, written in HTML 4.0, are compatible with any common web browser.

**Fig 1:**
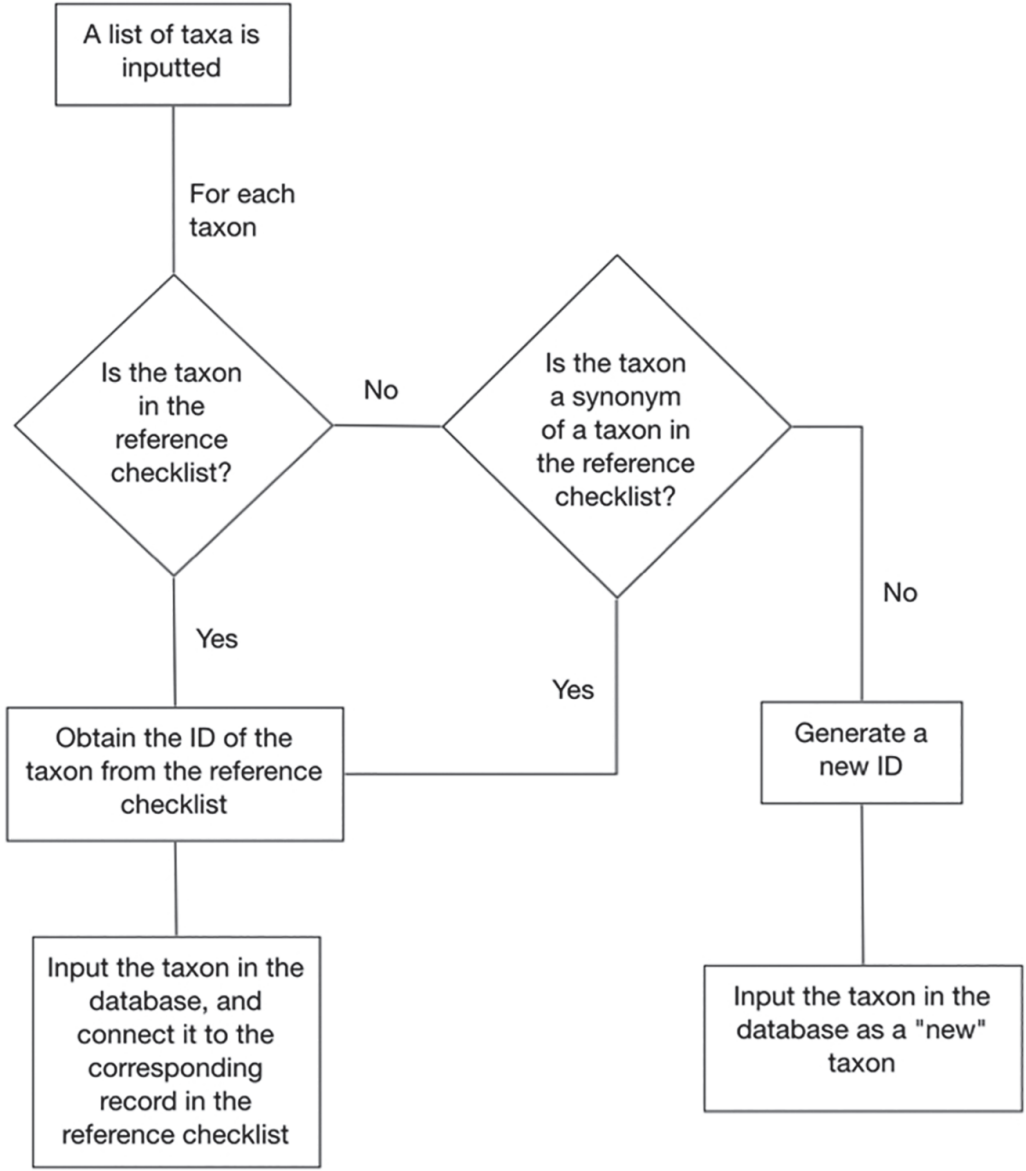
Input and storage of taxon names in the database of a portal. Once a list of names is inputted, the software checks whether the names match accepted names in the reference checklist, and stores them with their IDs in the checklist and in the database of morphological traits. Non-matching names are reported, and can be manually related to accepted taxa when they are synonyms, or inputted as “new taxa”.

The creation of a portal starts from the local checklist, which is stored in a database. The portal is built around it, following these main steps (see Results section for more details):

- Connection with the national checklists. This step is mandatory to harmonize nomenclature, and to prepare the list for the following steps of the process. All taxa which are not present in the national checklist (either as accepted names or synonyms) are highlighted in the process, and must be harmonized on a one by one basis by an expert. Future nomenclatural changes in the national checklist are automatically implemented in the portals. The process begins when a local checklist is fed into the system. This is done manually by selecting the taxa from a list of ca 30000 plant names stored in the database of morphological traits. Missing names can be added as new records. After the input (Fig. 1) the system automatically detects names which match accepted names in the reference checklist, and inputs them in the system with the corresponding unique identifier (ID) from the checklist. If a name is not recognised, it is reported to the user. These names can be manually related to accepted taxa when they are synonyms, or inputted as “new taxa” when they are not present in the reference checklist (e.g. in the case of newly discovered alien taxa or of newly described species). Since the list is a selection of records in the database of morphological traits, each name is also connected with the relative ID. The two IDs are used in retrieving information while querying a portal.
- Generation of a data table with several morphological characters for each taxon. This step involves an automatic connection to the morphological databases of Project *Dryades*, which currently host data for ca. 30000 taxa known to occur in Italy and Europe (ca. 8500 infrageneric taxa of vascular plants). This is made by a software which, for each taxon, selects the states of a number of easily observable characters (39 for vascular plants), which are used in the multi-access query interface. These data are stored together with IDs, taxon and family names into a data table. Potentially, any other database of morphological traits can be used in the process. However, in this case, the multi-entry interface software should be adapted to the new traits, if they differ from those which are currently in use.

In the querying process, the software works on these data up to the results and/or taxon pages, or when an identification key is invoked. When displaying the results page, or when building a taxon page, the software obtains information and media from:

- the image archives of project *Dryades*, which currently host ca. 175000 digital images of several groups of organisms (vascular plants, lichens, mosses, fungi, etc.). The archives return the URLs of the images, together with the related metadata, which are displayed both in the results pages and/or in the taxon pages.
- the database of Italian vernacular names, that currently hosts ca. 100000 names. Vernacular names are displayed in the taxon pages only.
- the reference checklist, to obtain data about the distribution of the taxon, and any other information, incl. notes/descriptions. The tuning of the system requires knowledge on the structure of the reference checklist, and of its data. In the case of Italy, the distributional information is used to generate a distribution map as well. These data are displayed in the taxon pages only.

The connection with the different archives and the portals is made by sending the taxon name as a key-value pairs (KVP) request. The archives return the data encoded into eXtenible Markup Language (XML) files, the portals being capable of decoding the XML files, and of displaying the data.

### Identification keys

An identification key can be invoked whenever the portal returns a list of taxa after using the multi-access query interface. While the connection to a key is not mandatory for a portal, it greatly improves its outreach. Presently, all the portals we published on the Web are linked to an identification key.

**Fig 2:**
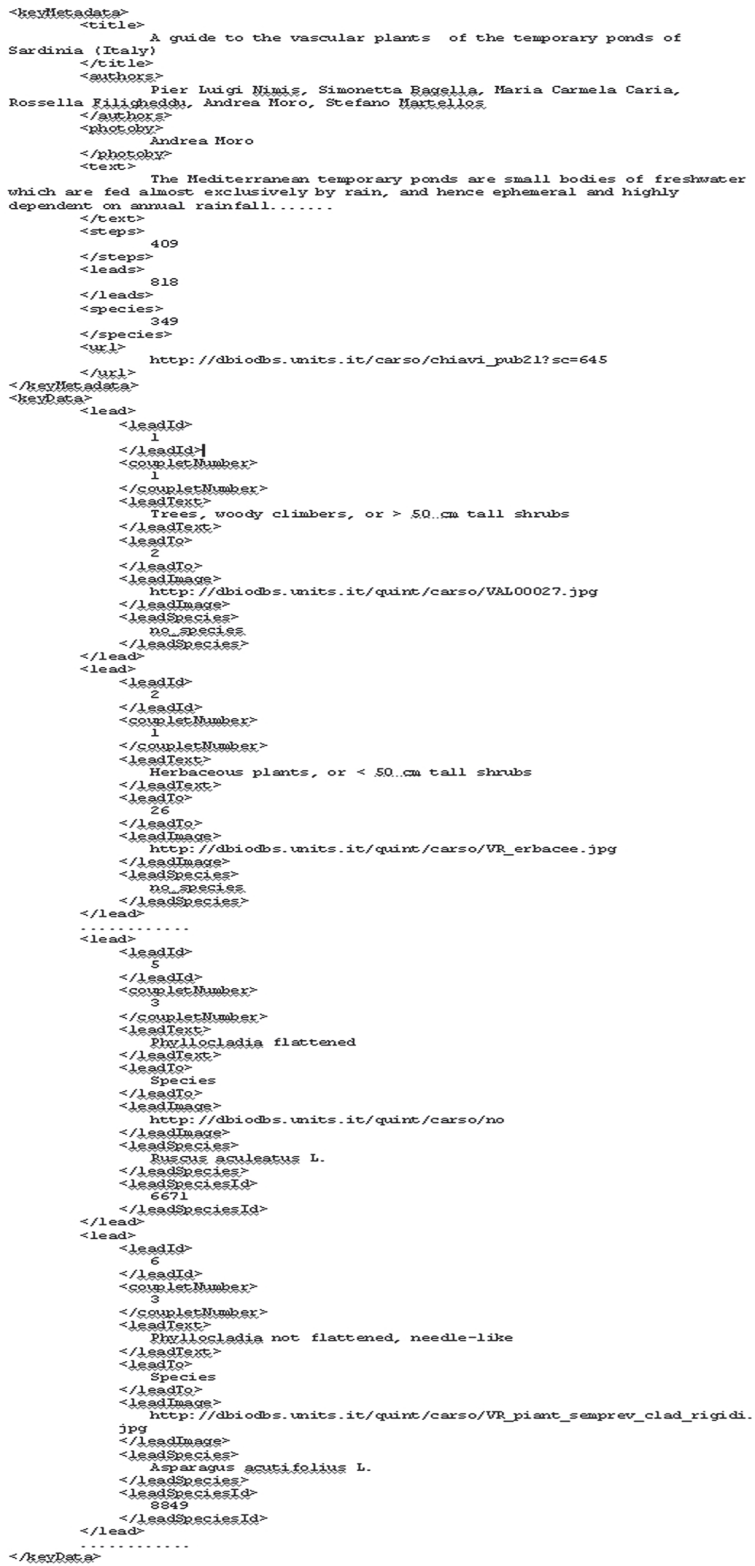
Example of a XML file containing a dichotomous key. The element keyMetadata contains title, authors, introduction, number of taxa and of steps. The element keyData stores the key, and is made of as many lead elements as the leads of the key. Each lead element can contain the following nested elements: leadId, coupletNumber, leadText, leadTo, leadImage, leadSpecies, leadSpeciesId

The dichotomous key is sent to the portal software encoded into an XML file. An example of the XML code is given in Fig. 2. The first element (keyMetadata) contains metadata such as title, authors, a brief introduction, number of taxa and of steps, etc., which are decoded, and stored in an array.

The actual key is stored in the keyData element. This is made of as many lead elements as the leads of the key. Several elements are nested inside the lead element:

- leadId—the unique ID of the lead,
- coupletNumber—the number of the couplet of which the lead is part,
- leadText—the text of the lead, which normally contains a sentence made of a character and one of its states, but also a more complex ensemble of characters and states,
- leadTo—the couplet to which the lead brings to, or the string "species", when the lead brings to a taxon,
- leadImage—the URL of the image which illustrates the text of the lead, if available,
- leadSpecies—the name of the taxon to which the lead brings, or the string "no_species" if the lead brings to a couplet,
- leadSpeciesId—the ID of the taxon in the database of morphological traits from which the key is derived. This element is present only when the lead brings to a taxon.

**Fig 3:**
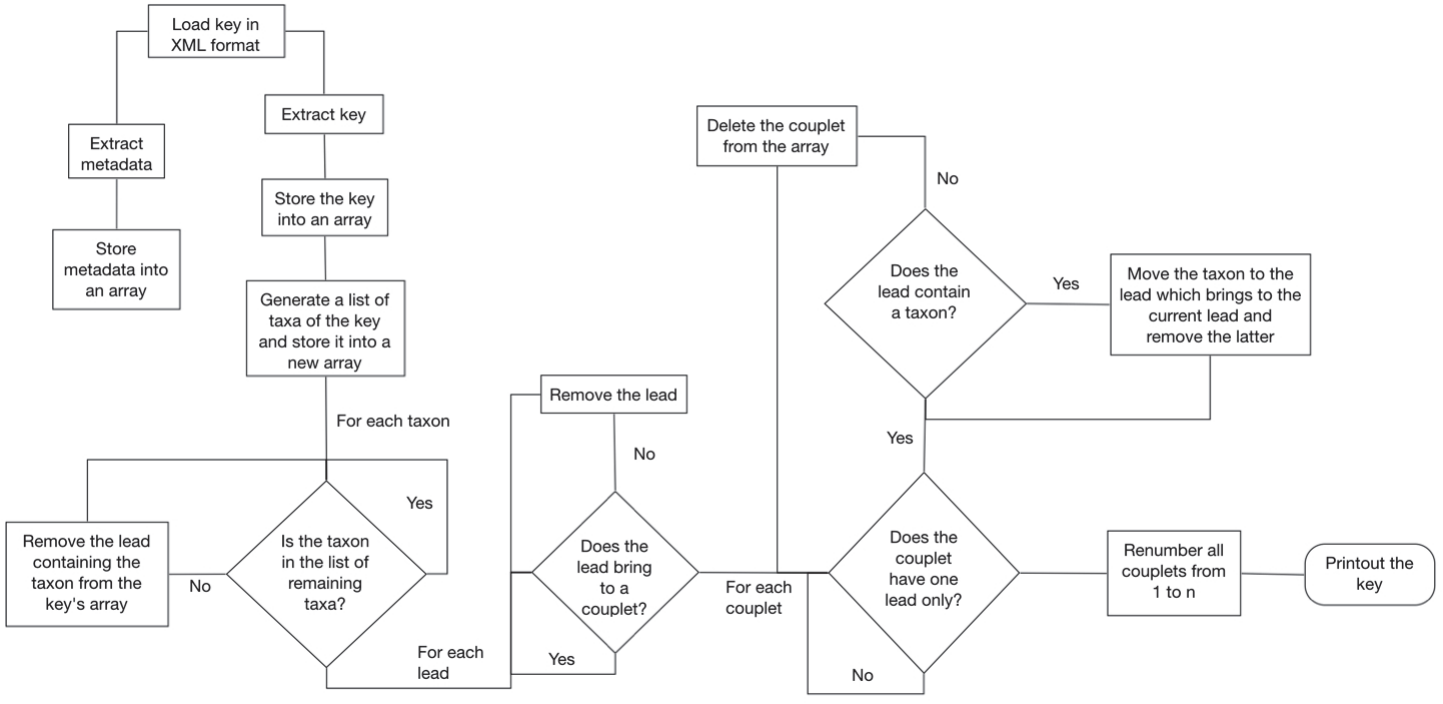
Reducing a key. The key array is compared with the list of remaining taxa by using the IDs, maintaining in the key array the useful leads only, and building a new, smaller dichotomous key.

The keyData element is decoded and stored in an array as well. Since the whole key normally contains far more taxa than the list obtained by querying the multi-entry interface, the software starts a reduction process (Fig. 3). The key array is compared with the list of remaining taxa by using the taxon IDs. This process removes from the array all the leads containing taxa which are not in the list. Then, through three different phases, the software purges the array from all the useless leads, and builds a new, smaller dichotomous key, which is printed out in the web browser of the user, enriched with illustrations and taxon pages.

The original dichotomous key is selected from the archive of *Dryades* at the moment of the creation of the portal, but the selection can be easily changed anytime. Potentially, any dichotomous key which can be exported in the XML format described above could be used in a portal, with an additional constraint: each taxon in the key must have the same ID it has in the database of morphological traits which is used in generating the portal.

Once connected to the system, a key can be automatically exported in static formats as well: for an example, see the Flora of the Southern Carnic Alps, which is available as a book [25], as a web portal (http://dryades.units.it/ampezzosauris_en/), in a version for CD-Rom, and as an app for mobile devices.

## Results: The New Portals

An example of a portal in English is that to the flora of temporary ponds of Sardinia (Italy), accessible at the address http://dryades.units.it/stagnisardi_en. The landing page lists the full name of the portal, the author(s), and a brief synopsis of its content. The menu gives access to several sections:

- information section, describing how the system works, and how to use it;
- survey area section, describing the survey area with texts and images;
- query section (described in detail below);
- checklist section, listing all taxa alphabetically by genus and species name, and providing direct access to their taxon pages;
- credits section, listing the institutions which contributed to the development of the portal.

**Fig 4:**
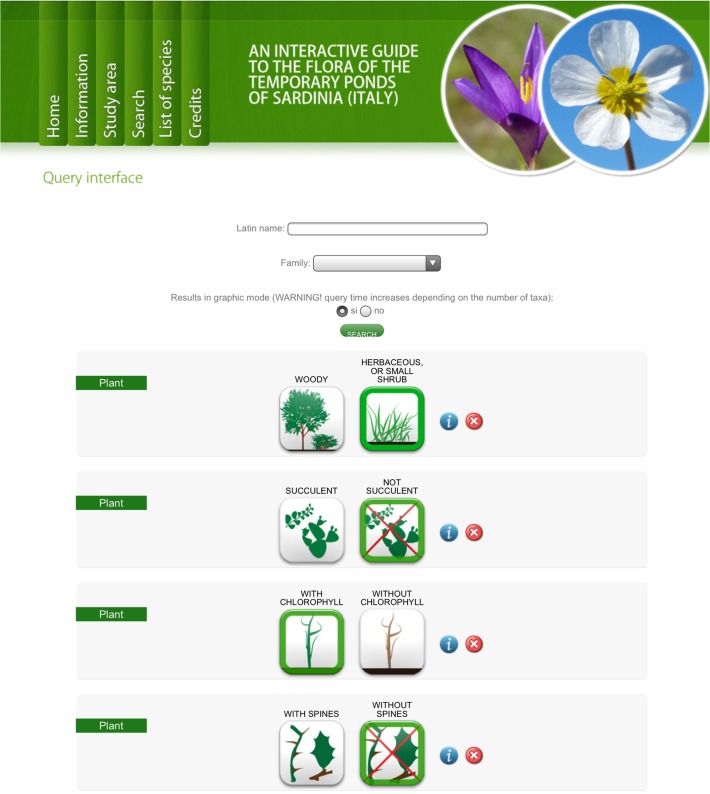
Multi-entry interface . Users can query the database by taxon name, family, and/or by a combination of several easily observable morphological characters. Each character state is displayed by icons, which remain highlighted when clicked.

**Fig 5:**
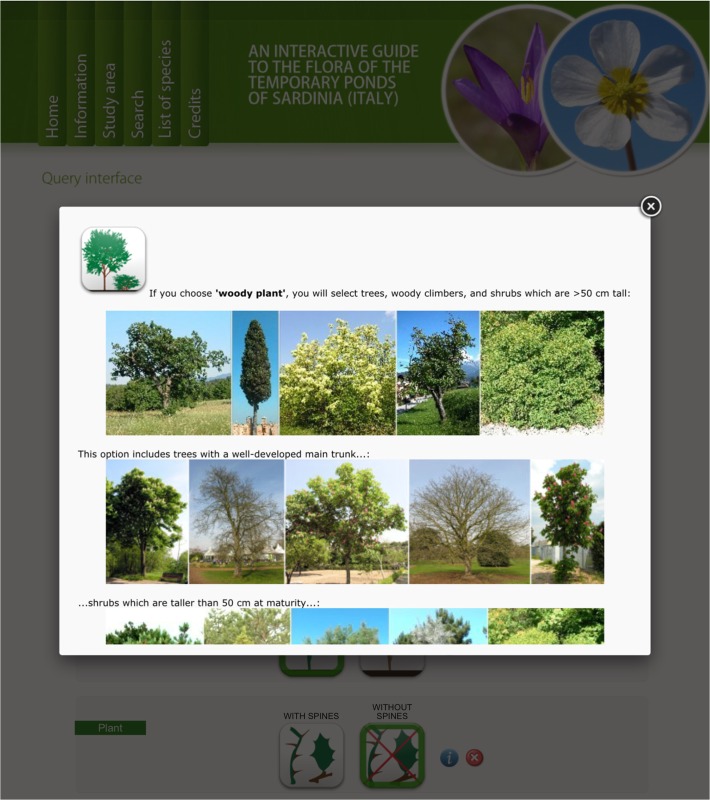
Popup window with information on a character.

**Fig 6:**
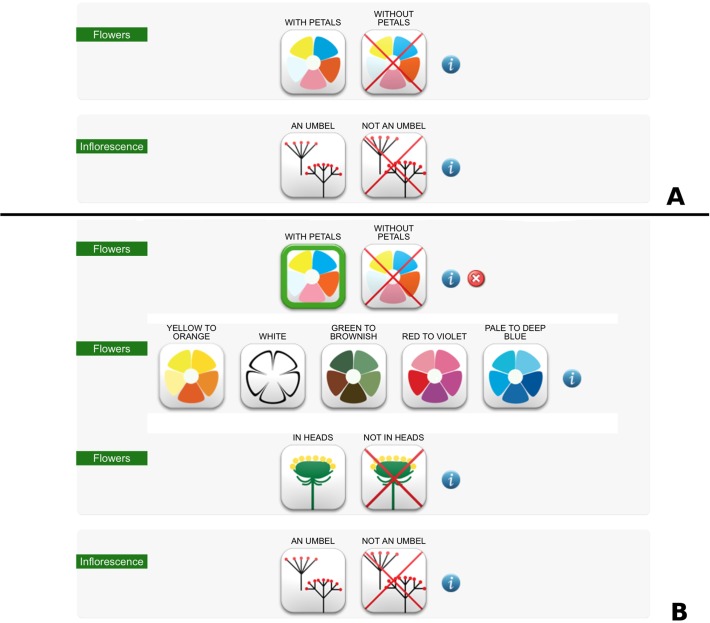
Some of the characters become accessible only when specific character states are selected. When selecting the state “with petals” for the character “Flowers” (a), the character "colour of petals" becomes usable (b).

**Fig 7:**
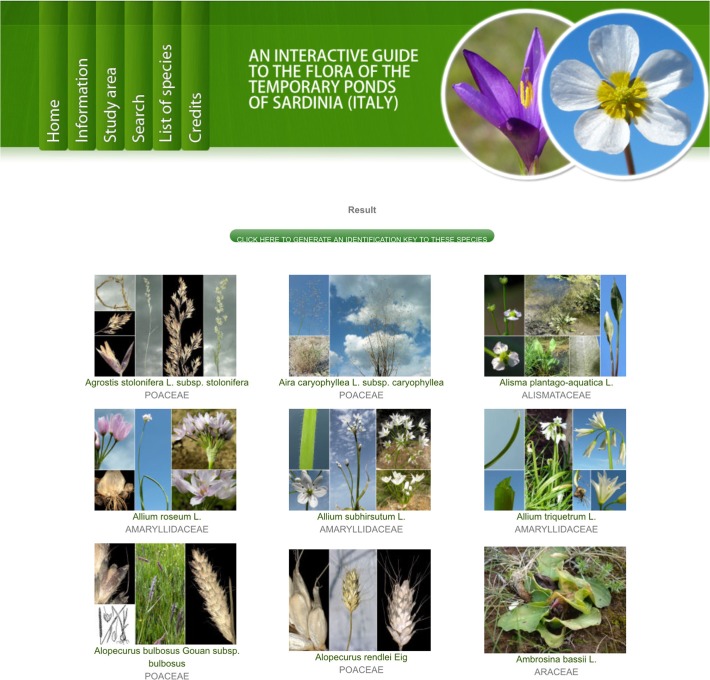
The multi-entry interface produces an illustrated gallery of taxa sharing the selected characters.

**Fig 8:**
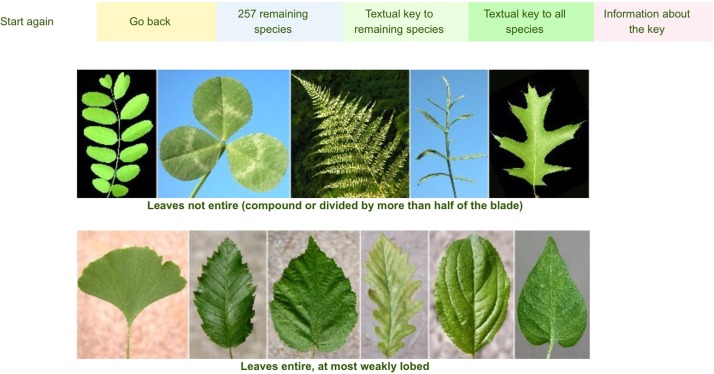
Digital dichotomous key. It includes only the taxa selected using the multi-entry interface.

**Fig 9:**
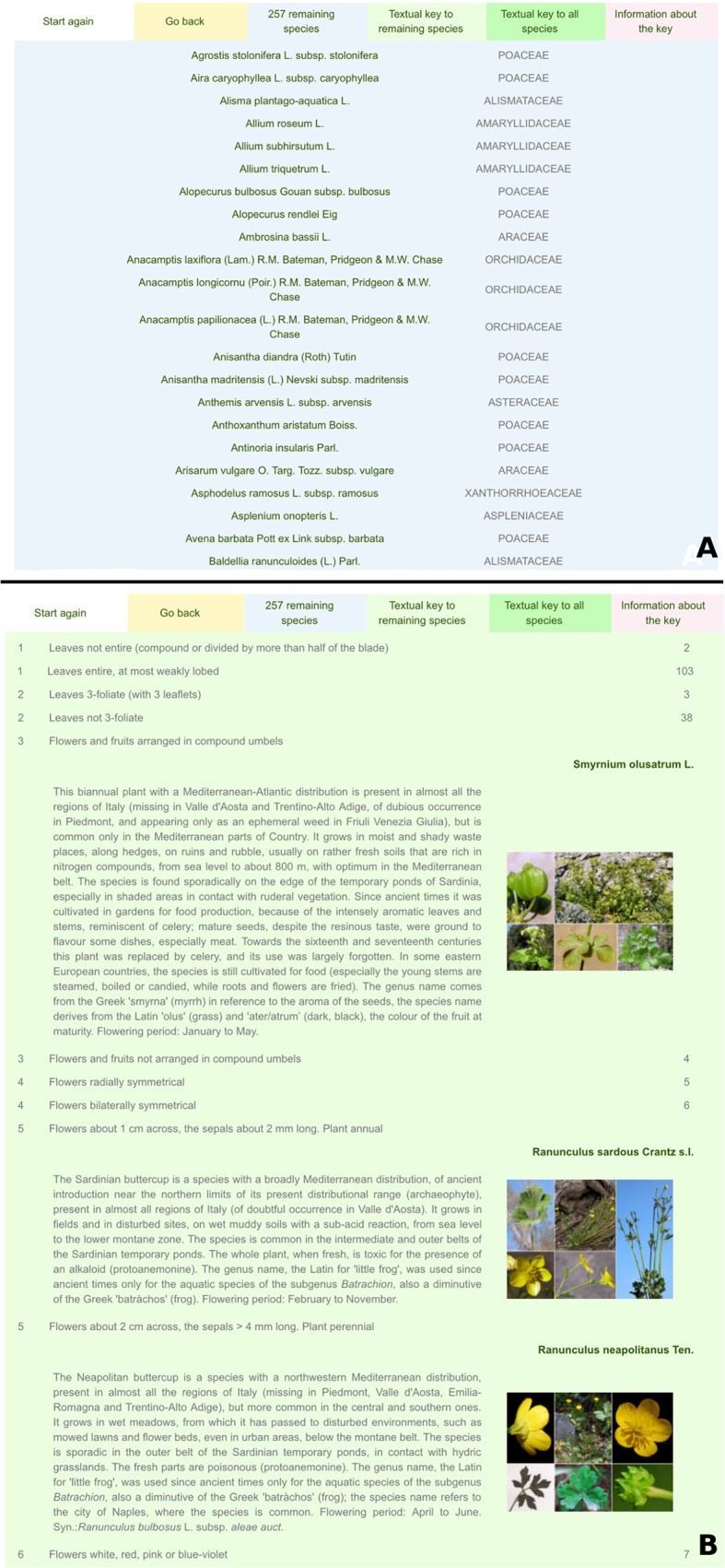
At each step of the identification process, users can list out the remaining taxa (a) or print an illustrated textual key (b).

**Fig 10:**
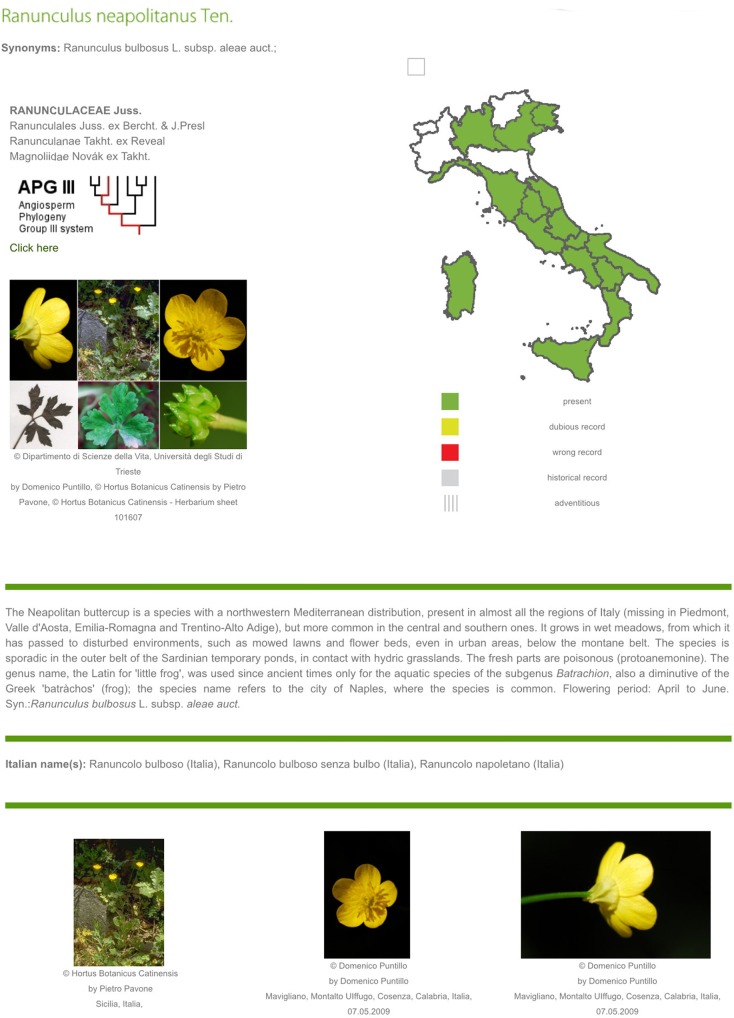
Taxon page. It lists scientific name, synonyms (when present), family and its position in the APG III system, an image, a distribution map in the regions of Italy, vernacular names, a note, and a gallery of images.

The identification system consists of two query interfaces:

1) The first is a multi-entry interface (Fig. 4) where users can specify a set of easily observable characters, and/or query the database by taxon name and family. The result is a photo gallery of all plants that possess the specified characters. Each character state is displayed by icons, which remain highlighted when clicked. A popup window (Fig. 5) with information on each character is accessible by clicking on the question mark button on the right side. Some of the characters become accessible only when specific character states are selected, e.g. the colour of petals becomes usable only when the character “flowers with petals” is selected (Fig. 6A-B).

2) The second interface, which is activated only after using the first one, is a richly illustrated dichotomous key which includes only the taxa selected using the multi-entry interface (Fig. 7). At each step of the identification process (Fig. 8), users can list out the remaining taxa (Fig. 9A), or print an illustrated textual key (Fig. 9B). By clicking on a taxon name, the corresponding taxon page is shown (Fig. 10), which displays:

- Scientific name,
- Synonyms,
- Family,
- Position of the family in the APG III system,
- An image, which is often a mosaic of images showing the most important characters of the taxon,
- A distribution map in the regions of Italy, generated automatically on the basis of information from the national checklist,
- Italian vernacular names,
- A note including: general distribution, Italian distribution, local distribution, ecology, uses, etymology, life-form, and flowering period,
- A gallery of all of images available in the archives of project *Dryades*, with their metadata.

Twenty-three portals are currently available in four languages (English, German, Italian and Slovenian), exposing on the Web the local checklists of several areas in Italy and in Slovenia (for a complete list see http://dryades.units.it/portali/?procedure=lista). Other portals will be published in the next future, to include local floras of other National and Regional Parks and Reserves, as a new mean to promote the knowledge of their biodiversity heritage.

## Discussion

The use of digital resources for storing and managing biodiversity data is no longer limited to an academic audience. It can be also relevant to laypersons like amateurs, tourists, or citizen scientists, who can access, and eventually share, new knowledge. Most of the biodiversity-related resources published on the Web are potentially accessible to a wide audience, but their usability is often poor. While an expert may profit from simple lists of names, laypersons may require that names are automatically linked to other resources. Identification tools, in particular, should be designed in such a way as to be usable, as much as possible and feasible, also by persons without a specific training in Systematics. Exposing scientific information to citizens can help in involving them in citizen science campaigns, e.g. for achieving a more detailed floristic exploration of a territory, or to monitor the changes in species distribution and composition in the short-, medium- and long-period. The importance of the involvement of citizens in understanding and monitoring biodiversity, as well as their participation in the development of effective environmental policies, has been clearly expressed by the European Commission, in the document "Establishing Horizon 2020" (EU Regulation no. 1291/2013), giving the address for all the future researches for the next 10 years.

One of the most relevant differences between our portals and similar online resources, e.g. that for the flora of Switzerland, an app for mobile devices developed on the basis of the “Flora Helvetica” [26], or the Flora of China online (http://flora.huh.harvard.edu/china/), is the fact that the underlying dichotomous keys do not derive from previously existing, paper published, 'classical' keys, but are automatically generated by a computer, trying to give higher weight to characters that can be easily appreciated by laypersons. Furthermore, the choice to concentrate on local checklists makes the use of these keys easier, because they include only the species which are present in a rather restricted area.

Since 2003, the resources we published online have reached ca. nine millions page loads (an average of ca. 2500 page loads per day). After the publication of the first portals, at the end of 2012, the number of page-loads increased substantially, and the number of unique visitors is rapidly approaching 1.000.000. Since the first portal was published, at the end of 2013, the portals were accessed ca. 210.000 times. In the week 15–21 September 2014 we scored ca. 30000 page loads and 5600 unique visitors. These figures, that are constantly increasing with the publication of new portals, demonstrate how wide is the potential audience of biodiversity-related resources, when these are designed for a non-academic public. Positive feedback was received from several categories of users, from amateurs to school teachers, the latter being especially eager to use these resources in their teaching scenarios. Another interesting advantage of our portals is related to their promotional value for parks and local authorities [18].

Presently, it is possible to generate a portal for vascular plants from almost any local checklist from Italy and Central Europe. The same approach can be easily followed for other organisms, see e.g. the Information System on Scyphozoa, Cubozoa and Staurozoa (http://dryades.units.it/jelly/), or the multi-entry interface in the key to the Parmelioid lichens of Thailand (http://dbiodbs.units.it/carso/chiavi_pub21?sc=507).

*Ad hoc* dichotomous keys can be also generated and connected to the portal, but this process does involve an active exchange of information with the author(s) of the checklist. In fact, the portals can potentially be generated from any list of taxa, with the following contraints:

1. There must be a reference checklist in digital format working as a nomenclatural backbone.
2. There must be a database of morphological traits which can be used with the multi-entry query interface,
3. There must be a dichotomous key, exportable in the XML format described above (optional),

The possibility of rapidly transforming thousands of local checklists already available in the literature into interactive identification portals is likely to bring to greater awareness of biological diversity by citizens, and hence to their deeper involvement in its conservation. In order to facilitate this process, we have decided to share with the Scientific Community a critical mass of resources:

1) The availability of pictures is one of the main bottlenecks for the creation of a portal. Our archive of digital pictures of vascular plants presently consists of 143689 images of 15641 infrageneric taxa. Starting from October 2014, more than 90% of the pictures from our archive (i.e. those taken by personnel of the University of Trieste) are being released under a Creative Commons license (CC BY-SA 3.0), and can be thus freely used by anybody. The pictures are downloadable in high resolution, with their metadata, from our 'Plantfinder' site (http://dbiodbs.units.it/carso/cercapiante01). These images have also been aggregated in the Europeana databases (http://www.europeana.eu). In the course of 2015, also the digital pictures of lichens and other organisms will be released under the same license.

2) The usability of dichotomous keys is greatly enhanced by the availability of illustrations for characters and characters states. For vascular plants, our archive includes more than 1800 composite images referring to characters, which are also made available to the scientific community under a CC BY-SA license since 2015.

3) A CSV file (S1 File) containing ca. 8400 infrageneric taxa of vascular plants, with the characters used in the multi-entry query interface of our portals, is made available under a CC BY-SA license in the additional material attached to this article. A list of the characters and their states is provided as well (S2 File).

## Supporting Information

S1 File Records which are used in the development of the portals to vascular plants. For each record, the state of 38 characters is given as a number. The information is provided in CSV format.
(CSV)

S2 File Characters and their states. For each character, states are numbered from 1 to n. The numbers of the states are used to describe the records of the S1 File. The information is provided in CSV format.
(CSV)
